# MAFF mitigates oxidative stress and pyroptosis in cardiopulmonary bypass-induced myocardium injury

**DOI:** 10.3389/fphys.2025.1516167

**Published:** 2025-07-31

**Authors:** Lei Yuan, Duo Wang, Yale Su, Long Yuan, Mixia Li, Dongdong Zheng, Cuilin Zhu, Hulin Piao, Yong Wang, Zhicheng Zhu, Dan Li, Tiance Wang, Kexiang Liu

**Affiliations:** ^1^ Department of Cardiovascular Surgery, The Second Norman Bethune Hospital of Jilin University, Changchun, Jilin, China; ^2^ Third Clinical Medical School, School of Acupuncture, Moxibustion and Tuina, Henan University of Chinese Medicine, Zhengzhou, Henan, China

**Keywords:** cardiopulmonary bypass, myocardial injury, bioinformatics, MAFF, reactive oxygen species, pyroptosis

## Abstract

**Background:**

Cardiopulmonary bypass (CPB) remains an indispensable technique for open-heart surgery; however, it induces systemic inflammation and oxidative stress, leading to myocardial cell damage and compromised prognosis. Optimizing myocardial protection during CPB remains a critical objective. This study aimed to identify potential therapeutic targets for myocardial protection during CPB.

**Methods:**

We performed weighted gene co-expression network analysis (WGCNA) on previously published datasets (GSE12486, GSE132176, GSE14956, and GSE38177) to identify CPB-related hub genes. An *in vitro* model of oxidative stress was established using H_2_O_2_-treated H9C2 cardiomyocytes to validate these hub genes. Through systematic validation, we identified the most representative hub gene. Subsequent functional studies, including gene knockdown and overexpression experiments, were conducted to elucidate its role and underlying mechanisms in oxidative stress-induced cardiomyocyte injury.

**Results:**

Integrated bioinformatics analysis and experimental validation identified MAFF as the most differentially expressed hub gene between pre- and post-CPB conditions. In the oxidative stress model, MAFF overexpression demonstrated cardioprotective effects by maintaining cell viability, significantly reducing reactive oxygen species (ROS) accumulation in both cytoplasm and mitochondria, and attenuating pyroptosis-mediated cell death.

**Conclusion:**

Our findings demonstrate that MAFF exerts protective effects against oxidative stress-induced cardiomyocyte injury, positioning it as a promising therapeutic target for myocardial protection. These results provide novel insights into optimizing postoperative recovery and improving clinical outcomes for patients undergoing CPB-assisted cardiac surgery.

## 1 Introduction

Cardiopulmonary bypass (CPB) is a cornerstone technology in modern cardiac surgery, enabling complex intracardiac operations under controlled hemodynamic conditions (Borst and Gründeman, 1999; Evora et al., 2016). However, despite its indispensable clinical utility, CPB triggers a series of non-physiological responses that include systemic inflammation, oxidative stress, and ischemia-reperfusion injury (Ferreira et al., 2023; Becker et al., 2021). These pathological alterations may give rise to myocardial injury, thereby triggering complications such as low cardiac output syndrome and arrhythmia, which exert a detrimental impact on patient outcomes (Robert et al., 2015; Kochar et al., 2022; Ma et al., 2018). The underlying mechanisms involve excessive accumulation of reactive oxygen species (ROS), mitochondrial dysfunction, and activation of inflammatory signaling cascades, which collectively result in cardiomyocyte damage (Baufreton et al., 2006; Ng and Wan, 2012; Lonati et al., 2018; Wen et al., 2019).

Although numerous strategies—such as advanced perfusion systems, optimized cardioplegia, and pharmacological interventions—have been proposed to attenuate CPB-related myocardial injury, clinical efficacy remains limited (McRae and de Perrot, 2018; Zakkar et al., 2015a). Recent studies suggest that oxidative stress and pyroptosis, a highly inflammatory form of programmed cell death mediated by NLRP3 inflammasome activation, play pivotal roles in CPB-induced cardiac damage (Zhou et al., 2024; Zakkar et al., 2015b). However, the upstream transcriptional regulators that orchestrate these processes remain poorly understood, and effective molecular targets for therapeutic intervention are still lacking. Therefore, identifying key transcriptional regulators underlying CPB-induced myocardial injury and developing biomarkers and therapeutic targets are of critical importance for myocardial protection and improved clinical prognosis.

To address this knowledge gap, we employed an integrated multi-omics strategy—combining large-scale transcriptomic profiling, weighted gene co-expression network analysis (WGCNA), and mitochondrial gene screening—to identify key regulators involved in CPB-induced myocardial injury. This approach identified MAFF as a potential core gene linked to oxidative stress and immune pathways. MAFF, a small MAF, is a basic region leucine zipper (bZIP)-type transcription factor composed of a DNA binding domain and a leucine zipper domain necessary for dimerisation. MAFF can mediate both transcriptional activation or repression by forming heterodimers with other bZIP transcription factors (von Scheidt et al., 2021). Previous studies have demonstrated that inhibition of MAFs results in reduced mRNA levels of several antioxidant genes; additionally, MAFs exhibit the capacity to significantly inhibit apoptosis (Bensellam et al., 2015; Wang et al., 2021). We subsequently confirmed its functional involvement through *in vitro* models of oxidative stress and ischemia-reperfusion, demonstrating that MAFF regulates redox balance, preserves mitochondrial function, suppresses inflammatory signaling and pyroptosis. These findings suggest that MAFF may represent a novel regulatory node in CPB-related myocardial injury and a promising target for therapeutic cardioprotection.

## 2 Materials and methods

### 2.1 Data sources and mitochondrial gene acquisition

Gene expression data were acquired from the Gene Expression Omnibus (GEO) database. Preoperative and postoperative gene expression data were acquired from datasets GSE12486, GSE132176, GSE14956, and GSE38177. All datasets comprised RNA samples, with GSE12486, GSE14956, and GSE38177 generated using the Affymetrix HG-U133 Plus 2.0 Array (GPL570), while GSE132176 utilized the Affymetrix HT HG-U133+ PM Array (GPL13158). Cross-platform normalization was implemented via the sva R package, followed by differential expression analysis using the limma R package with significance thresholds set at |logFC| > 0.5 and P < 0.05. Details of the datasets are presented in Table 1. Multi-dataset normalization was performed using the sva package, and differentially expressed genes (DEGs) were identified using the limma package, applying criteria of absolute log fold change (logFC) > 0.5 and P-value <0.05. Mitochondria-associated DEGs were identified through systematic annotation against Gene Ontology (GO) terms containing “mitochondria” (Thomas et al., 2022). The complete list of mitochondria-associated DEGs was presented in Supplementary Table S1.

**TABLE 1:** Included datasets and samples in this study.

ID (GSE)	Total sample (cases)	Pre-CPB (cases)	Post-CPB (cases)	Sample types	Platforms (GPL)
12486	10	5	5	RNA	570
132176	40	20	20	RNA	13158
14956/38177	12	6	6	RNA	570

### 2.2 Identification of Characterized genes for CPB-induced myocardial injury

WGCNA employs systems biology principles to detect clusters of highly correlated genes and associate these modules with clinical phenotypes (Langfelder and Horvath, 2008). In this study, we implemented WGCNA on merged, normalized expression data from datasets GSE12486, GSE132176, GSE14956, and GSE38177, consolidated into a unified training cohort. Pre-processing excluded genes with missing values or zero standard deviation across samples to ensure analytical robustness. Network construction partitioned genes into discrete modules based on expression patterns. Subsequent correlation of module eigengenes with myocardial injury traits identified modules showing the strongest phenotypic associations. The highest-correlating module, exhibiting maximal statistical significance (P-value minimization), was prioritized for downstream analysis as it likely harbors pathologically relevant genes underlying CPB-associated myocardial injury.

### 2.3 Identification of key mitochondrial-related genes for CPB-induced myocardial injury

Venn R package was employed to intersect the DEGs, characterized genes, and mitochondrial genes to identify core mitochondrial-related genes in CPB. Expression trend graphs and receiver operating characteristic (ROC) curves for these core genes in both the control and CPB groups were plotted with the pROC package.

### 2.4 Functional enrichment analysis

GO and Kyoto Encyclopedia of Genes and Genes (KEGG) enrichment analyses were conducted to further elucidate the biological functions and significance of the DEGs, characterized genes, and core genes related to myocardial injury CPB-induced myocardial injury. ggplot2, cluster Profiler, org. Hs.e.g.,.db, and enrich plot packages were utilized for the enrichment analysis. Criterion of significance was P-value <0.05 (Kanehisa and Goto, 2000).

### 2.5 Immune infiltration analysis

CIBERSORT algorithm was implemented to quantify 22 immune cell subsets in pre-/postoperative samples from the training cohort (permutations = 1000, P < 0.05) (Newman et al., 2015) (Supplementary Table S2). Spearman correlation analyses in R established associations between candidate hub genes and immune cell infiltration patterns, visualized via ggplot2. Network analyses using netET and linkET packages delineated functional interactions between mitochondrial hub genes and immune response pathways.

### 2.6 Cell culture and treatment

H9C2 cells were purchased from the Cell Bank of the Institute of Biochemistry and Cell Biology (Shanghai, China), were cultured in Dulbecco’s Modified Eagle Medium (DMEM) (Gibco, United States) supplemented with 10% fetal bovine serum (Gibco, United States), and 1% (v/v) penicillin/streptomycin, at 37°C in a humidified incubator containing 5% CO2 and 95% air. H9C2 cells were induced with H_2_O_2_ (350 μM) for 2 h at 90% confluency to establish the cardiomyocyte injury model for the subsequent experiments (Li et al., 2022).

### 2.7 Quantitative real-time PCR (RT-qPCR)

TRIzol reagent (Ambion, United States) was employed for RNA extraction. The concentration (ng/μL) of total RNA was quantified and evaluated for purity with a Nanodrop ultraviolet spectrophotometer (Thermo Fisher Scientific, United States). RNA was reverse-transcribed into cDNA using first-strand cDNA synthesis kit (TransGen Biotech, China), following manufacturer protocols. SYBR Green qPCR master mix was used for RT-qPCR analysis, which was run on Bioer LineGene 9,600 Plus RT-qPCR system (Bioer Technological Co., Ltd., Zhejiang, China). GAPDH was used as the control. The comparative Ct method (2^−ΔΔCT^) was used to analyze data. The sequences of primers for RT-qPCR are as follows: GAPDH forward:5′-GGAGCGAGATCCCTCCAAAAT-3′; reverse: 5′-GGCTGTTG-TCATACTTCTCATGG-3′; MAFF forward:5′-CTCGAGATGTCTGTGGATCC-3′; reverse: 5′-GAATTCCTAGGAGCAGGA- 3′; NLRP3 forward:5′-GATCTTCGCTG-CGATCAACAG-3′; reverse: 5′-CGTGCATTATCTGAACCCCAC- 3′.

### 2.8 Western blot (WB) analysis

Total protein was extracted with Mammalian Total Protein Extraction Kit (TransGen Biotech, China) following manufacturer protocols. Protein concentrations were determined using the BCA Protein Assay kit (Thermo Fisher Scientific). Protein lysates were then run on a 10% or 12% SDS-PAGE gel and transferred onto a PVDF membrane (Millipore, United States). Thereafter, the membrane was blocked with blocking buffer (Genefist, China) and incubated with primary bodies overnight at 4° C. MAFF (ABclonal, China), NLRP3 (Zenbio, China), GSDMD-N (Abcam, United States), cleaved caspase-1 (C-caspase-1) (Abcam, United States), cleaved IL-1β (C-IL-1β) (Abcam, United States), GAPDH (Abcam, United States), and β-tubulin (Zenbio, China) were examined. The membranes were washed 5 times by 5 min each in tris-buffered saline with 0.1% Tween-20 (TBS-T). Horseradish peroxidase (HRP)-conjugated secondary antibodies (Goat anti-rabbit IgG H&L (HRP), Abcam, United States) were applied to the membranes for 50 min at room temperature. After three 5-min washes in TBS-T, the blots were developed with ECL substrate (Zenbio, China) and visualized with the chemiluminescence imaging system (Beijing JUNYI Electrophoresis Co., Ltd., China). Protein expression levels were quantified via densitometry using ImageJ software.

### 2.9 Cell viability assay

Cell viability was assessed using the Cell Counting Kit-8 (TransGen Biotech, China) following manufacturer protocols. H9C2 cells were seeded in 96-well plates at the density of 5,000 cells per well, then incubated for 24 h. The appropriate induction conditions were applied based on the experimental groups, with three replicate wells for each group. Thereafter, 10 μL of CCK reagent was added to each well and incubated for 2 h at 37°C. The absorbance was measured using a microplate reader (Bio-Tek, United States) to measure OD450. The measurements are normalized to control groups.

### 2.10 Lactate dehydrogenase (LDH) activity

LDH activity was assessed with LDH assay kit (Beyotime, China) following manufacturer protocols. H9C2 cells were seeded in 96-well plates at the density of 5,000 cells/well, then incubated for 24 h. For each sample, the working buffer consisted of INX, enzyme solution, and lactic acid solution. A total of 120 μL cell culture supernatant and 60 μL of working buffer were mixed and incubated at 37°C for 30 min. Finally, the absorbance was measured at 490 nm using a microplate reader. Data were then exported and analyzed.

### 2.11 Analysis of mitochondrial respiratory function

Mitochondrial energetics were assessed with an oxygen consumption rate (OCR) assay kit (Bestbio). H9C2 cells were seeded in 96-well plates at the density of 5,000 cells per well, then incubated for 24 h. After treatment, 96 μL DMEM and 4 μL BBoxiProbe R01 were added per well, followed by 30-min incubation at 37°C. Oxygen-sealing reagent (100 μL/well) was applied prior to fluorescence measurement (excitation/emission: 460/603 nm) on a Bio-Tek microplate reader, with readings acquired at 10-min intervals. OCR values were calculated using manufacturer-defined algorithms.

### 2.12 Evaluation of Mitochondrial Permeability Transition Pore opening

mPTP opening in H9C2 cells was assessed by the Calcein‐AM/CoCl_2_ quenching technique with mPTP Assay Kit (Beyotime, China). In brief, H9C2 cells were cultured on coverslips in 24-well plates at a density of 2 × 10^4^ cells/well, then incubated for 24 h. After pretreatment, the cells were loaded with calcein-AM and a cytosolic calcein fluorescence quencher (CoCl_2_) in the dark at 37°C for 30 min. Mitochondrial calcein fluorescence images were acquired with a fluorescent microscope (Olympus, Japan).

### 2.13 Mitochondrial membrane potential (MMP)

JC-1 staining (Beyotime, China) was used to measure MMP following manufacturer protocols. The bright red fluorescence indicates J-aggregates and the green fluorescence indicates J-monomers. In brief, H9C2 cells were cultured on coverslips in 24-well plates at a density of 2 × 10^4^ cells/well, then incubated for 24 h. Cells were loaded with JC-1 in serum-free medium in the dark at 37°C for 20 min after pretreatment, and then washed twice with JC-1 staining buffer. Fluorescence was observed with a fluorescent microscope (Olympus, Japan).

### 2.14 Cell transfection

Cardiomyocyte transfection was performed using Lipofectamine 3,000 (Invitrogen) per manufacturer guidelines. Cells were plated in 6-well plates (1.5 × 10^5^ cells/well) and cultured overnight. Plasmid DNA (2 μg/well; MAFF overexpression or control vectors) and Lipofectamine 3,000 (7.5 μL/well) were diluted in Opti-MEM medium (250 μL), incubated for 20 min at room temperature, and applied to cells. After 4–6 h incubation (37°C, 5% CO_2_), transfection medium was replaced with fresh DMEM. Cells were harvested 24–48 h post-transfection for downstream analyses, with transfection efficiency validated via RT-qPCR and immunoblotting. MAFF-targeting siRNA sequences: sense 5′-CCAGCAAAGCUCUAAAGAUTT-3′; antisense 5′-AUCUUUAGAGCUUUGCUGGTT-3′. Scramble control: sense 5′-UUCUCCGAACGUGUCACGUTT-3′; antisense 5′-ACGUGACACGUUCGGAGAATT-3′.

### 2.15 Detection of ROS

An ROS assay kit (Beyotime, China) was used to detect the accumulation of ROS in H9C2 cells and MitoSOX red (Beyotime, China) staining was performed for the detection of mitochondrial superoxide production, according to the manufacturer’s instructions. In brief, H9C2 cells were cultured on coverslips in 24-well plates at a density of 2 × 10^4^ cells/well. Cells were loaded with DCFH-DA (10 μM) and mitoSOX red (5 μM) in serum-free medium in the dark at 37°C for 30 min after pretreatment, and then washed three times with PBS. Fluorescence was observed with a fluorescent microscope (Olympus, Japan).

### 2.16 TUNEL staining

Cell apoptosis was evaluated by terminal deoxynucleotidyl transferase-mediated dUTP nick-end labeling (TUNEL) staining (Beyotime, China). Cardiomyocytes were seeded at the density of 75,000/well in 12-well culture plates, then incubated for 24 h. After treatments, cells were washed with PBS for 3 times and then fixed with 4% paraformaldehyde. For permeabilization, the cells were incubated with PBS with 0.3% Triton-X100 at room temperature for 5 min. After 3 washes, TUNEL stains were added and incubated at 37°C for 1 h. Thereby, after 3 washes, the cells were counterstained with DAPI (Solarbio, China). Then the cells were washed 3 times and mounted with anti-fade media. Fluorescent microscopy and image acquisition were conducted with Olympus IX71 (Olympus, Japan).

### 2.17 Hypoxia/re-oxygenation model (H/R model)

H9C2 cardiomyocytes were replaced with FBS-free and glucose-free DMEM and exposed to a hypoxic environment of 95% N2 and 5% CO_2_ at 37°C for 4 h, while the control groups were cultured under normal conditions. H9C2 cells were then replaced with ordinary medium and placed in an ordinary incubator for 2 h to simulate reoxygenation injury (Son et al., 2020).

### 2.18 Statistical analysis

The experiments were performed in triplicate. The statistics and graph generation were conducted using GraphPad Prism 8. The statistical distinctions between the groups were assessed by using a two-tailed Student’s t-test. P < 0.05 was considered to be statistically significant.

## 3 Results

### 3.1 Identification of DEGs for CPB-induced myocardial injury

**FIGURE 1:**
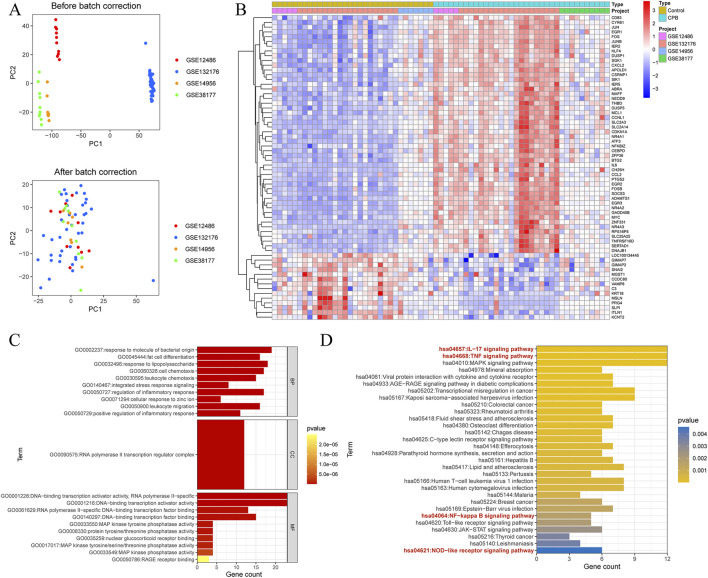
Identification of DEGs for CPB-induced myocardial injury. **(A)** Before and after the standardization of GSE12486 GSE132176, GSE14956 and GSE38177. **(B)** Heatmap of DEGs. **(C)** GO enrichment analysis of the DEGs. **(D)** KEGG pathway enrichment analysis of the DEGs. DEGs, differentially expressed genes; CPB, cardiopulmonary bypass; GSE, Gene Expression Omnibus Series; GO, Gene Ontology; KEGG, Kyoto Encyclopedia of Genes and Genes.

After obtaining the expression matrix for the data of CPB-induced myocardial injury, normalization was performed (Figure 1A). Subsequent differential analysis conducted using R identified a total of 117 DEGs associated with CPB-induced myocardial injury (Supplementary Table S3), then a heatmap of these genes was generated (Figure 1B). Finally, GO and KEGG enrichment analysis was used to investigate DEGs. GO enrichment analysis suggested that a majority of genes are involved in inflammatory response, integrated stress response signaling, leukocyte migration and chemotaxi, and other related processes (Figure 1C; Supplementary Table S4). KEGG enrichment analysis indicated that these genes are associated with NOD-like receptor signaling pathway, NF-kappa B signaling pathway, IL-17 signaling pathway and TNF signaling pathway (Figure 1D; Supplementary Table S5).

### 3.2 Identification of Characterized genes for CPB-induced myocardial injury

**FIGURE 2:**
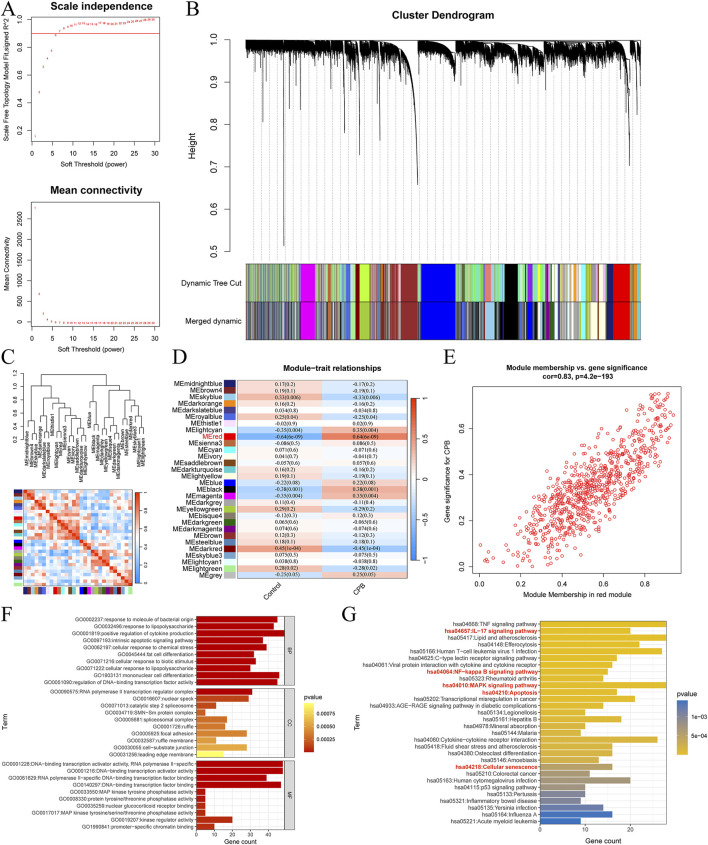
Identification of Characterized genes for CPB-induced myocardial injury. **(A)** Soft threshold analysis. **(B)** Merge similar threshold modules. **(C)** Gene modules related to CPB-induced myocardial injury. **(D)** Heatmap of correlations between different gene modules and traits, with p. adj values in parentheses and correlation coefficients outside parentheses for different modules. **(E)** Red module showing the strongest correlation. **(F)** GO enrichment analysis of the red module genes. **(G)** KEGG pathway enrichment analysis of the red module genes. CPB, cardiopulmonary bypass; GO, Gene Ontology; KEGG, Kyoto Encyclopedia of Genes and Genes.

WGCNA was performed on the data of CPB-induced myocardial injury, using a threshold of 6 for module division (Figure 2A). Similar modules were merged (Figure 2B), resulting in 30 gene modules related to CPB-induced myocardial injury (Figures 2C,D), with the red module showing the strongest correlation (R = 0.83, P = 4.2e-193, Figure 2E). A total of 755 genes were obtained, with the remaining module genes listed in Supplementary Table S6. Finally, GO and KEGG enrichment analysis was used to investigate these characterized genes. GO enrichment analysis suggested that a majority of genes are involved in cytokine production, mononuclear cell differentiation, cellular response to chemical stress, intrinsic apoptotic signaling pathway and other related processes (Figure 2F; Supplementary Table S7). KEGG enrichment analysis indicated that these genes are associated with NF-kappa B signaling pathway signaling pathway, IL-17 signaling pathway, apoptosis, MAPK signaling pathway, cellular senescence (Figure 2G; Supplementary Table S8).

### 3.3 Identification and assessment of core genes for CPB-induced myocardial injury

**FIGURE 3:**
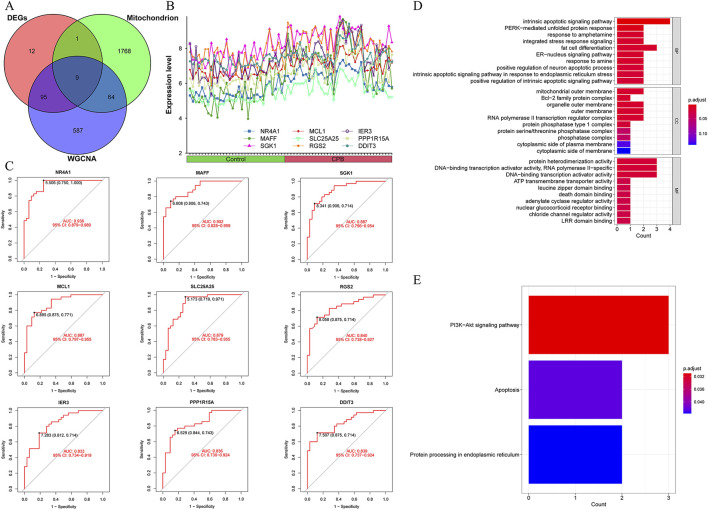
Identification and assessment of core genes for CPB-induced myocardial injury. **(A)** Vene map of DEGs, mitochondrial genes and WGCNA results. **(B)** Expression curves of core genes. **(C)** ROC curves of core genes. **(D)** GO enrichment analysis of the core genes. **(E)** KEGG pathway enrichment analysis of the core genes. CPB, cardiopulmonary bypass; DEGs, differentially expressed genes; WGCNA, Weighted Gene Co-Expression Network Analysis; ROC, receiver operating characteristic; GO, Gene Ontology; KEGG, Kyoto Encyclopedia of Genes and Genes.

The intersection of DEGs, characterized genes, and mitochondrial genes for CPB-induced myocardial injury yielded 9 core genes (Figure 3A). The expression trends of these 9 core genes were plotted, revealing that all are highly expressed due to CPB-induced myocardial injury (Figure 3B). ROC curve analysis indicated that these nine genes possess good diagnostic value (Figure 3C). Finally, GO and KEGG enrichment analysis was used to investigate these core genes. GO enrichment analysis suggested that a majority of genes are involved in integrated stress response signaling, intrinsic apoptotic signaling pathway, PERK-mediated unfolded protein response, and other related processes (Figure 3D; Supplementary Table S9). KEGG enrichment analysis indicated that these genes are associated with apoptosis and protein processing in endoplasmic reticulum (Figure 3E).

### 3.4 Immune infiltration analysis of core genes for CPB-induced myocardial injury

**FIGURE 4:**
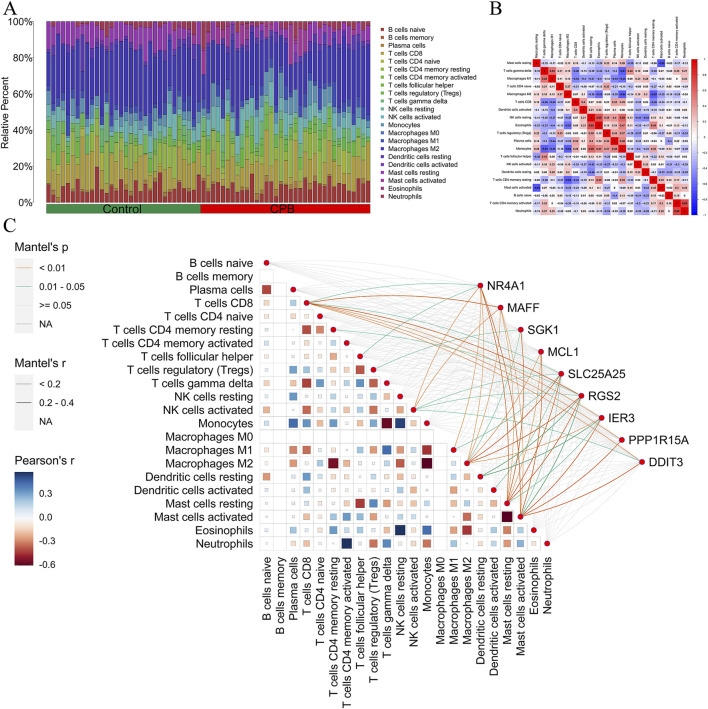
Immune infiltration analysis of core genes for CPB-induced myocardial injury. **(A)** The composition ratio between different immune cells in the control group and CPB group. **(B)** Heatmap of different immune cells. **(C)** Map of correlation between core genes and immune infiltration. CPB, cardiopulmonary bypass.

To further explore the immune microenvironment of CPB-induced myocardial injury and whether these 9 core genes are involved in immune regulation of CPB-induced myocardial injury, we first plotted the immune landscape for the control and CPB groups (Figure 4A) and examined the correlations among immune cells in the CPB group (Figure 4B). Finally, we investigated the relationship between the 9 core genes and immune infiltration (Figure 4C). The results indicate a correlation between core genes and T cells as well as resting mast cells, highlighting the involvement of these genes in immune regulation of CPB-induced myocardial injury.

### 3.5 The expression of MAFF is significantly enhanced in the oxidative stress model and plays a protective role in maintaining cell viability and function

**FIGURE 5:**
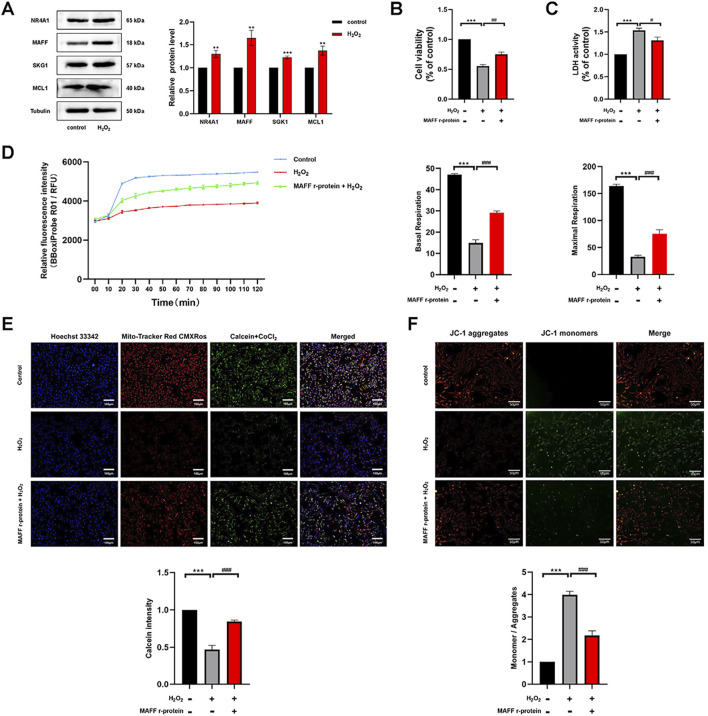
The expression of MAFF is significantly enhanced in the oxidative stress model and plays a protective role in maintaining cell viability and function. **(A)** The protein expression levels of NR4A1, MAFF, SKG1, MCL1 were analyzed by Western blot. **(B)** CCK-8 assay. **(C)** The level of LDH release from H9C2 cells. **(D)** OCR in H9C2 cells, Basal respiration and Maximal respiration of H9C2 cells. **(E)** mPTP opening levels in H9C2 cells. Triple staining of Calcein (green), Mito-Tracker (red) and Hoechst 33342 (blue). Scale bar 100 µm. **(F)** JC-1 was used to examine the mitochondrial membrane potential. JC-1 aggregates (red) and JC-1 monomers (green). Scale bar 50 µm. Data were presented as mean ± SD (n = 3). (^**^p < 0.01 vs. control group, ^***^p < 0.001 vs. control group, ^#^p < 0.05, ^##^p < 0.01, ^###^p < 0.001 MAFF r-protein + H_2_O_2_ group vs. H_2_O_2_ group). CCK-8, cell-counting kit 8; LDH, Layered Double Hydroxide; OCR, Oxygen Consumption Rate; mPTP, Mitochondrial Permeability Transition Pore; MAFF r-protein, Recombinant Mouse Transcription factor MAFF.

Based on the results of our bioinformatics analysis, we identified a total of nine core genes, including NR4A1, MAFF, SKG1, and MCL1, that were significantly upregulated in cardiomyocytes following extracorporeal circulation and exhibited strong correlations with immune cell infiltration patterns (Figures 3, 4). Bioinformatics analysis identified nine CPB-related differentially expressed hub genes. The top 30% of these genes, which exhibited the strongest association with CPB based on ROC curve analysis—NR4A1, MAFF, SGK1, and MCL1—were selected for further validation through *in vitro* experiments (Figure 3C). As a proof of concept, we further investigated the expression profiles of these four genes in H9C2 cells under oxidative stress conditions. An *in vitro* oxidative stress model was established in H9C2 cells using H_2_O_2_ to simulate the oxidative stress conditions experienced by cardiomyocytes during extracorporeal circulation (Li et al., 2022; Canty et al., 1999). Western blot analysis revealed that MAFF exhibited the most significant upregulation under oxidative stress (*p* = 0.003) (Figure 5A), and therefore, it was selected for subsequent investigation. To preliminarily investigate the effect of MAFF on cardiomyocytes under oxidative stress, H9C2 cells were pretreated with Recombinant MAFF (MAFF r-protein) for 24 h, after which an *in vitro* oxidative stress model was established. Cell viability was assessed using the CCK-8 assay, and the release of LDH in the supernatant of H9C2 cells was measured. The results demonstrated that under oxidative stress conditions, cell viability decreased and LDH release increased. However, MAFF r-protein treatment increased cell viability (*p* = 0.001) (Figure 5B) and reduced LDH release (*p* = 0.013) (Figure 5C). Furthermore, the OCR assay revealed that H_2_O_2_ significantly decreased both basal respiration and maximal respiration in H9C2 cells compared to the negative control group. However, MAFF r-protein attenuated these effects, significantly elevating basal respiration and maximal respiration (*p* < 0.001) (Figure 5D). Additionally, compared to the negative control group, H_2_O_2_ significantly increased mPTP opening in H9C2 cells. MAFF r-protein attenuated this effect and reduced mPTP opening levels (Figure 5E). Moreover, JC-1 analysis was performed to assess MMP. The results revealed that the H_2_O_2_ group exhibited substantial MMP impairment compared to the negative control group, whereas the addition of MAFF r-protein significantly attenuated this MMP damage (Figure 5F). Based on these results, in the H9C2 cell model of oxidative stress, MAFF expression was markedly upregulated and demonstrated the most significant increase among the studied factors, playing a protective role in maintaining cell viability and function.

### 3.6 Downregulation of MAFF exacerbates oxidative stress and pyroptosis in H9C2 cells within the oxidative stress model

**FIGURE 6:**
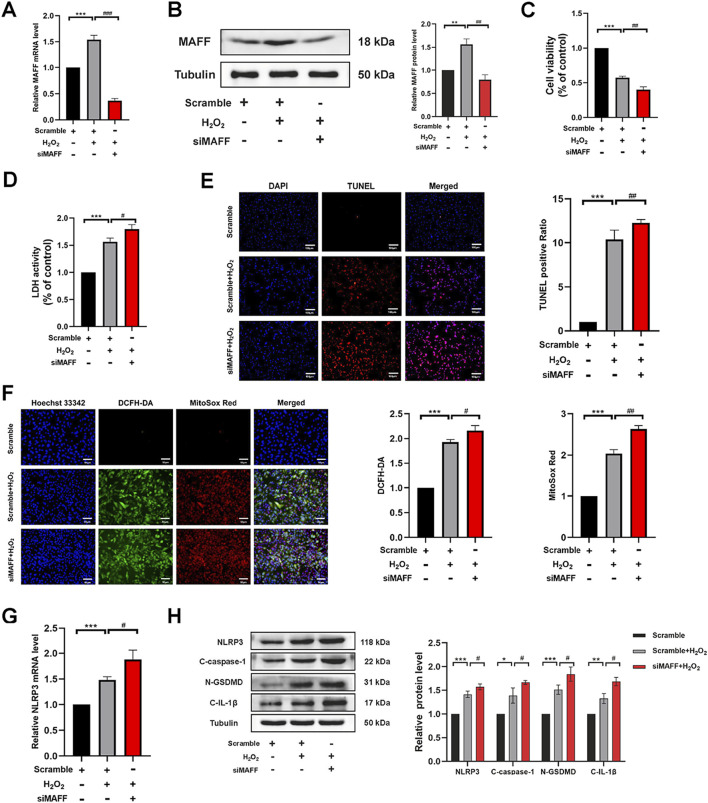
Downregulation of MAFF exacerbates oxidative stress and pyroptosis in H9C2 cells within the oxidative stress model. **(A)** RT-qPCR validates the MAFF downregulation efficiency. **(B)** Western blot validates the MAFF downregulation efficiency. **(C)** CCK-8 assay. **(D)** The level of LDH release from H9C2 cells. **(E)** TUNEL staining. Double staining of TUNEL (red) and DAPI (blue). Scale bar 100 µm. **(F)** DCFH-DA staining detecting cytoplasmic ROS generation and MitoSOX staining detecting mitochondrial ROS. Triple staining of DCFH-DA (green), MitoSOX (red) and Hoechst 33342 (blue). Scale bar 50 µm. **(G)** The mRNA expression level of NLRP3 was analyzed by RT-qPCR. **(H)** The protein expression levels of NLRP3, cleaved caspase-1, GSDMD-N, and cleaved IL-1β were analyzed by Western blot. Data were presented as mean ± SD (n = 3). (^*^p < 0.05, ^**^p < 0.01, ^***^p < 0.001 H_2_O_2_ group vs. Scramble group, ^#^p < 0.05, ^##^p < 0.01, ^###^p < 0.001 siMAFF + H_2_O_2_ group vs. H_2_O_2_ group). RT-qPCR, quantitative real-time PCR; CCK-8, cell-counting kit 8; LDH, Layered Double Hydroxide; TUNEL, transferase-mediated dUTP nick-end labeling; ROS, reactive oxygen species.

To further elucidate the role and underlying mechanisms of MAFF in oxidative stress-induced cardiomyocyte injury, we employed MAFF-targeted siRNA to suppress MAFF protein expression. The knockdown efficiency was validated by RT-qPCR (*p* < 0.001) (Figure 6A) and Western blot analysis (*p* = 0.001) (Figure 6B). Following transfection, H9C2 cells were exposed to H_2_O_2_ for 2 h. CCK-8 and LDH assays indicated that downregulation of MAFF significantly decreased cell viability (*p* = 0.003) (Figure 6C) and enhanced LDH release (*p* = 0.018) (Figure 6D) compared to the positive control group. TUNEL staining further revealed an increased proportion of TUNEL-positive cells upon downregulation of MAFF relative to the positive control (Figure 6E). ROS detection assays demonstrated that downregulation of MAFF markedly elevated intracellular ROS and mitochondrial superoxide levels in H9C2 cells compared to the positive control (Figure 6F). RT-qPCR analysis showed that downregulation of MAFF significantly upregulated NLRP3 mRNA expression in H9C2 cells (*p* = 0.023) (Figure 6G). Additionally, Western blot analysis confirmed that downregulation of MAFF increased the protein levels of NLRP3 (*p* = 0.038), GSDMD-N (*p* = 0.034), C-caspase-1 (*p* = 0.044), and C-IL-1β (*p* = 0.011) in H9C2 cells compared to the positive control (Figure 6H). Taken together, these findings suggest that downregulation of MAFF exacerbates oxidative stress-induced cellular damage, oxidative stress, and pyroptosis in H9C2 cells.

### 3.7 Overexpression of MAFF alleviates oxidative stress and pyroptosis in H9C2 cells within the oxidative stress model

**FIGURE 7:**
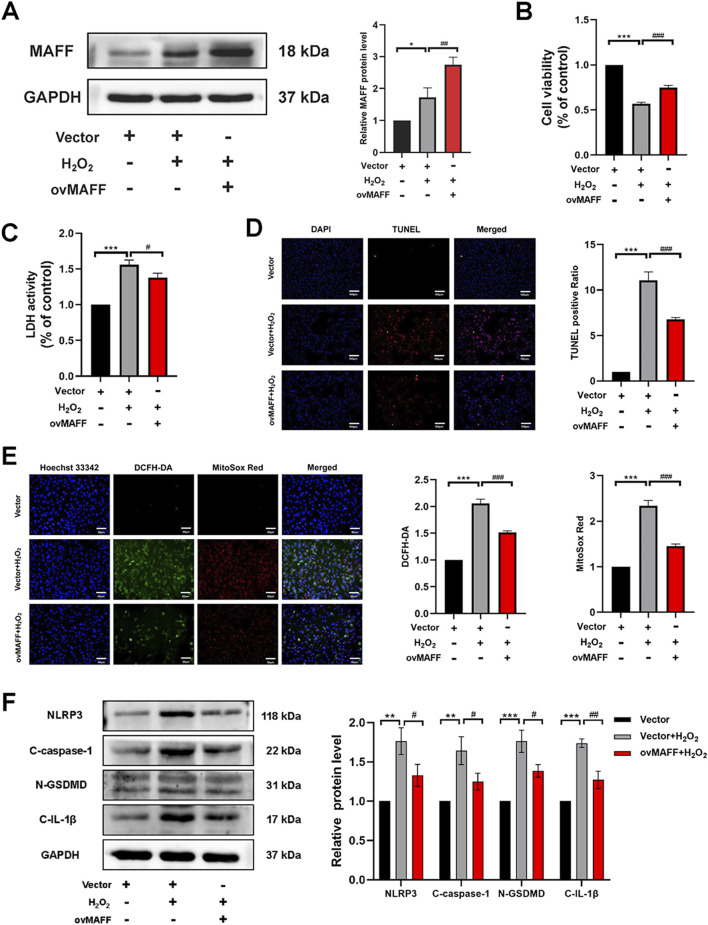
Overexpression of MAFF alleviates oxidative stress and pyroptosis in H9C2 cells within the oxidative stress model. **(A)** Western blot validates the MAFF overexpression efficiency. **(B)** CCK-8 assay. **(C)** The level of LDH release from H9C2 cells. **(D)** TUNEL staining. Double staining of TUNEL (red) and DAPI (blue). Scale bar 100 µm. **(E)** DCFH-DA staining detecting cytoplasmic ROS generation and MitoSOX staining detecting mitochondrial ROS. Triple staining of DCFH-DA (green), MitoSOX (red) and Hoechst 33342 (blue). Scale bar 50 µm. **(F)** The protein expression levels of NLRP3, cleaved caspase-1, GSDMD-N, and cleaved IL-1β were analyzed by Western blot. Data were presented as mean ± SD (n = 3). (^*^p < 0.05, ^**^p < 0.01, ^***^p < 0.001 H_2_O_2_ group vs. Vector group, ^#^p < 0.05, ^##^p < 0.01, ^###^p < 0.001 ovMAFF + H_2_O_2_ group vs. H_2_O_2_ group). CCK-8, cell-counting kit 8; LDH, Layered Double Hydroxide; TUNEL, transferase-mediated dUTP nick-end labeling; ROS, reactive oxygen species; ovMAFF, MAFF overexpression.

To further validate the cardioprotective effects and investigate the molecular mechanisms of MAFF in oxidative stress-induced cardiomyocyte injury and pyroptosis, H9C2 cells were transfected with either MAFF-overexpressing plasmids or empty vector controls. Following 48 h of transfection, MAFF overexpression efficiency was quantitatively assessed by Western blot analysis (*p* = 0.009) (Figure 7A). To induce oxidative stress, transfected cells were subsequently exposed to H_2_O_2_ for 2 h. Cellular viability assessment using CCK-8 assay revealed that MAFF overexpression significantly improved cell survival rate compared with vector-transfected controls (*p* < 0.001) (Figure 7B). Consistent with these findings, LDH release assay showed a marked reduction in cytotoxicity in MAFF-overexpressing cells (*p* = 0.026) (Figure 7C). Cell death was quantitatively analyzed by TUNEL staining, demonstrating a significant decrease in TUNEL-positive cells following MAFF overexpression (Figure 7D). To explore the antioxidant effects of MAFF, intracellular ROS and mitochondrial superoxide levels were measured, showing significant reduction in MAFF-overexpressing cells compared with controls (Figure 7E). Western blot analysis confirmed that MAFF overexpression significantly downregulated the protein expression levels of NLRP3 (*p* = 0.027), GSDMD-N (*p* = 0.016), C-caspase-1 (*p* = 0.03), and C-IL-1β (*p* = 0.003) in H9C2 cells compared with the positive control group (Figure 7F). These results provide compelling evidence that MAFF overexpression confers protection against oxidative stress-induced cellular damage in H9C2 cardiomyocytes, potentially through the regulation of ROS homeostasis and suppression of pyroptotic pathway activation.

### 3.8 MAFF mitigates oxidative stress and pyroptosis in H9C2 cells within the IRI model

**FIGURE 8:**
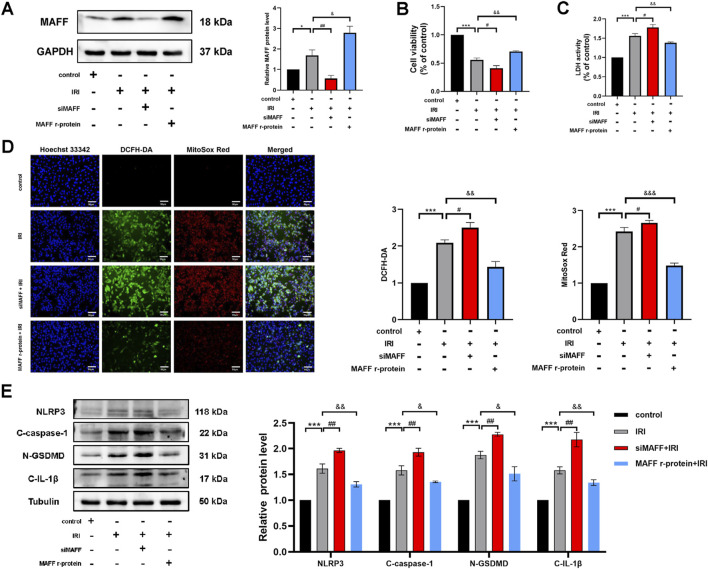
MAFF mitigates oxidative stress and pyroptosis in H9C2 cells within the IRI model. **(A)** The protein expression level of MAFF was analyzed by Western blot. **(B)** CCK-8 assay. **(C)** The level of LDH release from H9C2 cells. **(D)** DCFH-DA staining detecting cytoplasmic ROS generation and MitoSOX staining detecting mitochondrial ROS. Triple staining of DCFH-DA (green), MitoSOX (red) and Hoechst 33342 (blue). Scale bar 50 µm. **(E)** The protein expression levels of NLRP3, cleaved caspase-1, GSDMD-N, and cleaved IL-1β were analyzed by Western blot. Data were presented as mean ± SD (n = 3). (^*^p < 0.05, ^***^p < 0.001 control group vs. IRI group; ^#^p < 0.05, ^##^p < 0.01, IRI group vs. siMAFF + IRI group; ^&^p < 0.05, ^&&^p < 0.01, IRI group vs. MAFF r-protein + IRI group). IRI, Ischemia-Reperfusion Injury; CCK-8, cell-counting kit 8; LDH, Layered Double Hydroxide; ROS, reactive oxygen species; MAFF r-protein, recombinant MAFF protein.

CPB involves not only oxidative stress but also multiple pathophysiological mechanisms, including IRI and systemic inflammatory response. To further elucidate the role of MAFF in myocardial cell injury during CPB and its underlying mechanisms, we established an *in vitro* H9C2 cell model of IRI. In this model, MAFF protein expression was either knocked down or supplemented with MAFF recombinant protein. The efficiency of MAFF protein expression was validated by Western blot analysis (Figure 8A). CCK-8 assays revealed that compared to the negative control group, cell viability was reduced in the positive control group (*p* < 0.001) (Figure 8B). Furthermore, compared to the positive control group, downregulation of MAFF further decreased cell viability (*p* = 0.01), while the addition of MAFF recombinant protein improved cell viability (*p* = 0.002) (Figure 8B). LDH assays demonstrated that LDH release was increased in the positive control group compared to the negative control group (*p* < 0.001) (Figure 8C). Comparing to the positive control group, downregulation of MAFF further elevated LDH release (*p* = 0.017), whereas MAFF recombinant protein supplementation reduced LDH release (*p* = 0.009) (Figure 8C). ROS detection assays showed that intracellular ROS and mitochondrial superoxide levels were elevated in the positive control group compared to the negative control group (Figure 8D). Comparing to the positive control group, downregulation of MAFF further increased intracellular ROS and mitochondrial superoxide levels, while MAFF recombinant protein treatment decreased these levels (Figure 8D). Additionally, Western blot analysis confirmed that the expression levels of pyroptosis-related proteins, including NLRP3 (*p* < 0.001), GSDMD-N (*p* < 0.001), C-caspase-1 (*p* < 0.001), and C-IL-1β (*p* < 0.001), were increased in the positive control group compared to the negative control group (Figure 8E). Comparing to the positive control group, downregulation of MAFF further upregulated the expression of these pyroptosis-related proteins, while MAFF recombinant protein treatment downregulated their expression (Figure 8E). In summary, these results demonstrate that MAFF exerts a protective role in the IRI model by mitigating cellular injury, oxidative stress, and pyroptosis.

## 4 Discussion

CPB can induce myocardial injury, significantly impacting patient prognosis, yet effective molecular targets for therapeutic intervention remain lacking. Against this clinical backdrop, this study identified MAFF as a potential key regulator of CPB-associated cardiac damage through bioinformatics analysis. Notably, we discovered significant associations between MAFF and immune-related signaling cascades. Mechanistically, recombinant MAFF was demonstrated to enhance mitochondrial function and suppress intracellular/mitochondrial ROS generation *in vitro*, thereby preserving cardiomyocyte viability. Functional validation further revealed that MAFF alleviates oxidative stress and specifically inhibits NLRP3 inflammasome-mediated pyroptosis, establishing its cardioprotective effects. These findings provide experimental evidence confirming MAFF’s role as a regulator of oxidative stress and pyroptosis, providing experimental evidence that MAFF could be a potential therapeutic target for CPB-induced myocardial injury.

CPB was serving as a technical cornerstone in modern cardiothoracic surgery, but it can induce myocardial injury and adversely impacting patient prognosis (Salameh et al., 2020). Despite decades of exploration, efficient approaches to alleviate CPB-triggered myocardial injury remain elusive. Strategies such as optimized perfusion circuits, improved cardioplegia protocols, ischemic and anesthetic preconditioning, and mechanical circulatory support have shown partial benefits yet lack uniform curative efficacy across clinical settings. Recently, gene therapy has emerged as a theoretically attractive modality, offering the potential to enhance intrinsic myocardial defenses through targeted modulation of protective pathways. However, its clinical translation is still in its infancy. Moreover, a bunch of pharmacological candidates, including ROS scavengers, neutrophil adhesion inhibitors, and anti-inflammatory agents, have largely failed to demonstrate consistent improvements in patient outcomes across large-scale trials (Sabe et al., 2024). This therapeutic stagnation underscores a pressing demand to shift from empirical investigations toward mechanism-based interventions.

Recent studies have demonstrated that CPB induces the generation of significant levels of ROS (Zhao et al., 2022; Hatami et al., 2022; Hausenloy and Yellon, 2013; Cadenas et al., 2010). ROS mediates pyroptosis, an inflammatory programmed cell death mechanism implicated in myocardial pathophysiology (Zheng et al., 2022; Shi et al., 2021). This process is characterized by inflammasome activation, membrane pore formation, maturation and release of pro-inflammatory cytokines, such as IL-1β (Liu et al., 2023). NLRP3 inflammasome pathway represents the most characterized pyroptotic mechanism, comprising three core components: NLRP3 sensor protein, ASC adaptor, and caspase-1 effector (Ichinohe et al., 2013; Elliott and Sutterwala, 2015). ROS elevation directly upregulates NLRP3 expression, triggering inflammasome assembly (Mangan et al., 2018). In CPB contexts, ROS-driven NLRP3 inflammasome activation establishes a self-amplifying cycle of oxidative stress and pyroptosis, which not only amplifies tissue injury but also contributes to myocardial contractile dysfunction and adverse postoperative outcomes (Pearson et al., 2003; Subramanian et al., 2013). Therefore, targeted inhibition of the NLRP3 pyroptotic pathway presents a strategic therapeutic opportunity to enhance intraoperative cardioprotection. Based on these findings, identification of novel cardioprotective strategies targeting the NLRP3 pyroptotic pathway may provide innovative solutions to optimize myocardial protection during CPB.

This study systematically characterized molecular mechanisms underlying CPB-induced myocardial injury through multi-omics integration. Initial differential expression and WGCNA analyses identified 117 DEGs and 30 co-expression modules, respectively, with the red module highlighting inflammation-related pathways. Intersection of DEGs, module genes, and mitochondrial genes pinpointed nine core genes (e.g., upregulated in injury), showing strong diagnostic efficacy. Functional enrichment revealed their involvement in endoplasmic reticulum stress, apoptosis, and immune modulation, corroborating their pathological relevance. Notably, immune infiltration analysis demonstrated significant correlations between core genes and T-cell infiltration, suggesting their dual roles in myocardial damage and immune regulation. These findings bridge transcriptomic alterations with immunopathological processes, providing candidate biomarkers and therapeutic targets (e.g., NLRP3 inflammasome) for mitigating CPB complications. The convergence of oxidative stress, inflammatory cascades, and immune cell dysregulation aligns with clinical manifestations of post-CPB cardiac dysfunction, offering mechanistic insights for future interventional studies.

In this study, through integrative bioinformatics and experimental validation, we identified MAFF as a key transcriptional regulator involved in CPB-induced myocardial injury. Our analysis revealed that MAFF was among the most differentially expressed hub genes, showing strong associations with immune infiltration and oxidative stress pathways. Unlike other candidates such as SGK1 or MCL1, MAFF demonstrated a broader functional implication, potentially orchestrating upstream regulatory profiles related to redox homeostasis. Mechanistically, MAFF belongs to the small MAF (sMAF) transcription factor family, which lacks intrinsic transactivation domains but can functionally diversify by forming heterodimers with other basic leucine zipper-type transcription factors (von Scheidt et al., 2021; Blank, 2008; Katsuoka et al., 2016). This structural versatility allows MAFF to function as a transcriptional repressor as well as a co-activator in response to cellular stress. Accumulating evidence have emphasized the role of MAFF in modulating oxidation, inflammatory response, and apoptotic cascade, all of which are central to the pathogenesis of myocardial injury following CPB (Ferreira et al., 2023; Katsuoka et al., 2016).

In our study, among all differentially expressed genes observed, MAFF demonstrated the most pronounced upregulation under oxidative stress and the closest functional correlation with stress-related pathways. Compared to SGK1 and MCL1, which exert more circumscribed effects in ion regulation and anti-apoptotic cascade respectively, MAFF appears to function as a broader upstream modulator of stress-responsive transcriptional profiles. Notably, although NRF2 was not directly investigated in our study, previous studies have shown that sMAF proteins, particular MAFF, can heterodimerize with NRF2 to regulate antioxidant elements-driven genes (Katsuoka et al., 2005; Tanigawa et al., 2013; Hirotsu et al., 2012; Li et al., 2008). This interaction is critical for maintaining redox homeostasis and has been implicated in various models of oxidative injury. While the precise downstream mediators of MAFF in cardiomyocytes remain to be established, its known association with oxidative stress-regulatory networks underscores its therapeutic relevance. These findings underscore the therapeutic potential of MAFF in CPB-induced myocardial injury and reinforce the need for in-depth mechanistic exploration.

In this study, we demonstrated that MAFF confers robust protection against oxidative stress-induced cardiomyocyte injury. Both gain- and loss-of-function experiments confirmed that MAFF modulates key cellular processes, including the regulation of intracellular and mitochondrial ROS levels, preservation of mitochondrial function, and inhibition of pyroptotic cell death. These findings suggest that MAFF acts as a critical upstream modulator of redox homeostasis and inflammatory cell death under oxidative stress conditions. Given the central role of ROS and pyroptosis in the pathogenesis of CPB-induced myocardial injury, our investigations provide mechanistic insight into how MAFF may intervene at multiple points along this deleterious cascade. Pyroptosis, as a highly inflammatory form of programmed cell death, is primarily driven by activation of the NLRP3 inflammasome in response to cellular stressors, including ROS, mitochondrial dysfunction, and ion dysregulation (Park et al., 2023; Nguyen et al., 2023). Notably, our findings indicate that MAFF significantly suppresses ROS accumulation and, in turn, dampens NLRP3-mediated pyroptotic signaling. This dual regulatory role not only disrupts a key feed-forward loop in myocardial injury but also positions MAFF as a potential integrative node linking oxidative stress and innate immune activation.

However, our study has some inherent limitations. For instance, to investigate CPB-induced myocardial injury, we analyzed paired myocardial tissue samples collected before and after CPB from the same patients. This design minimized inter-individual variability and ensured that the observed gene expression changes were specifically attributable to CPB. However, the lack of normal myocardial tissue as a comparator may limit the ability to distinguish CPB-specific alterations from general stress-induced responses. Future studies incorporating normal cardiac samples could provide a more nuanced understanding of CPB-related transcriptomic changes. Additionally, given the multifactorial nature of CPB-induced myocardial injury, our *in vitro* model focused on H_2_O_2_-induced oxidative stress to capture one of the most critical early contributors to tissue damage. While this model does not fully recapitulate the complexity of CPB pathophysiology, its mechanistic specificity allows focused interrogation of redox-related injury. To partially address this limitation, we supplemented our analysis with an IRI model that better reflects clinical conditions. However, we acknowledge that both models remain simplified representations of the *in vivo* environment and cannot fully reproduce the intricate interplay of mechanical, inflammatory, and neurohumoral factors involved in CPB-induced myocardial injury. Moreover, although the H9C2 cell line is widely used for mechanistic studies in cardiovascular research, it does not fully mirror the electrophysiological, structural, or metabolic features of mature human cardiomyocytes. Therefore, the translational relevance of our findings should be interpreted with caution. Future validation using primary human cardiomyocytes or hiPSC-derived cardiomyocytes would enhance physiological relevance and clinical applicability. Furthermore, this study only provides preliminary verification of MAFF’s role in mitigating oxidative stress and ischemia-reperfusion injury. It did not investigate the underlying molecular mechanisms or specific targets of action, which represents a limitation of this work and serves as a key objective for subsequent research.

In summary, our findings have established a significant correlation between MAFF expression and myocardial injury during CPB, uncovering a novel cardioprotective pathway. The experimental results demonstrate that MAFF confers myocardial protection against oxidative stress-induced damage through two distinct yet interconnected mechanisms: substantial reduction of ROS accumulation in both cytosolic and mitochondrial compartments and significant attenuation of pyroptosis-mediated cell death. These mechanistic insights position MAFF as a promising therapeutic target for myocardial protection, providing valuable evidence for developing targeted cardioprotective strategies during CPB procedures. The clinical implications of these findings are particularly significant, as targeting MAFF-mediated pathways could potentially optimize postoperative recovery and improve clinical outcomes in CPB-assisted cardiac surgery patients.

## Data Availability

The original contributions presented in the study are included in the article/Supplementary Material, further inquiries can be directed to the corresponding author.

## Abbreviations

CPB, Cardiopulmonary bypass; ROS, Reactive oxygen species; mPTP, Mitochondrial Permeability Transition Pore; NLRP3, The nod-like receptor containing a pyrin domain 3; GSDMD, gasdermin-D; WGCNA, Weighted Gene Co-Expression Network Analysis; GO, Gene Ontology; KEGG, Kyoto Encyclopedia of Genes and Genes; GEO, Gene Expression Omnibus; DEGs, Differentially expressed genes; ROC, Receiver operating characteristic; DMEM, Dulbecco’s Modified Eagle Medium; qRT-PCR, Quantitative real-time PCR; WB, Western blot; CCK-8, Cell Counting Kit-8; LDH, lactate dehydrogenase; OCR, Oxygen Consumption Rate; MMP, Mitochondrial membrane potential; TUNEL, transferase-mediated dUTP nick-end labeling; MAFF r-protein, Recombinant Mouse Transcription factor MAFF; MAFF, MAF bZIP transcription factor F; IRI, ischemia-reperfusion injury; GSE, Gene Expression Omnibus Series; ovMAFF, MAFF overexpression.
